# A novel in vivo model of puncture-induced iris neovascularization

**DOI:** 10.1371/journal.pone.0180235

**Published:** 2017-06-28

**Authors:** Ophélie Beaujean, Filippo Locri, Monica Aronsson, Anders Kvanta, Helder André

**Affiliations:** Department of Clinical Neuroscience, Section of Eye and Vision, St. Erik Eye Hospital, Karolinska Institutet, Stockholm, Sweden; Children's Hospital Boston, UNITED STATES

## Abstract

**Purpose:**

To assess iris neovascularization by uveal puncture of the mouse eye and determine the role of angiogenic factors during iris neovascularization.

**Methods:**

Uveal punctures were performed on BalbC mouse eyes to induce iris angiogenesis. VEGF-blockage was used as an anti-angiogenic treatment, while normoxia- and hypoxia-conditioned media from retinal pigment epithelium (RPE) cells was used as an angiogenic-inducer in this model. Iris vasculature was determined in vivo by noninvasive methods. Iris blood vessels were stained for platelet endothelial cell adhesion molecule-1 and vascular sprouts were counted as markers of angiogenesis. Expression of angiogenic and inflammatory factors in the puncture-induced model were determined by qPCR and western blot.

**Results:**

Punctures led to increased neovascularization and sprouting of the iris. qPCR and protein analysis showed an increase of angiogenic factors, particularly in the plasminogen-activating receptor and inflammatory systems. VEGF-blockage partly reduced iris neovascularization, and treatment with hypoxia-conditioned RPE medium led to a statistically significant increase in iris neovascularization.

**Conclusions:**

This study presents the first evidence of a puncture-induced iris angiogenesis model in the mouse. In a broader context, this novel in vivo model of neovascularization has the potential for noninvasive evaluation of angiogenesis modulating substances.

## Introduction

In the eye, the iris is the most anterior portion of the uvea, which also constitutes the ciliary body and choroid. The iris epithelium is composed of two layers derived from the neuroectoderm during embryonic development, and is the most vascularized layer of the uvea. Iris arteries and veins originate from the outer limbus limits of the uvea and progress up on the inner iris bordered by the pupil. Plenty of anastomosis is found between arteries and veins [1], allowing nutrition and oxygen supply not only to the iris tissue but also to the anterior chamber of the eye, and therefore maintain intraocular homeostasis [2]. In mammals, the development of the eye is not final at birth. Mouse ocular vasculature development continues after birth with mice pups opening their eyes approximately 12.5 days after birth [3,4]. As such, induced angiogenesis models in the mouse eye, as is the example of oxygen-induced retinopathy (OIR), a model of retinopathy of prematurity, have been established based on these developmental characteristics [5].

Several events have been identified as cause of angiogenesis, both physiologic and pathogenic. Physiologic events of angiogenesis include wound healing, pregnancy, and uterine cycling [6], where inflammation, tissue growth or proliferation, and tissue remodeling occur. The breakdown of the extracellular matrix and basement membranes allows for new vessels to form, by proliferation of endothelial cells, and recruitment of pericytes as well as macrophages and other inflammatory cells [7]. In pathology, angiogenesis is activated by an imbalanced ratio between stimulatory and inhibitory factors, such as vascular endothelial growth factor (VEGF) and plasminogen activator inhibitor (PAI), as well as multiple inflammatory factors. With regards to the iris, angiogenesis is a complication of pre-existing ocular or systemic diseases [8]. Although in ocular diseases the focal neovascularization might be located in distinct tissues, rubeosis iridis (i.e. the clinical term for iris angiogenesis) originating from an increase of angiogenic factors in both anterior and posterior chambers of the eye has been associated with proliferative diabetic retinopathy (PDR) and neovascular glaucoma [9]. Moreover, due to transparency of the cornea, iris angiogenesis can be observed directly in clinical diagnostics, suggesting that animal models of iris angiogenesis could be easily evaluated and quantified in vivo by noninvasive methods [10].

Models of wound-healing have been associated with angiogenesis models [11] due to an induced increase in angiogenic factors. The present study is based on a mouse model of puncture-induced neovascularization of the iris. Punctures are performed posterior to the limbus wounding the uvea in order to induce the formation of new blood vessels in the iris. Vascular loops could be observed in punctured eyes and could be associated with an increase in vascular sprouting of the iris. Molecular evaluation of the punctured eyes revealed an increase in angiogenesis-related factors, particularly the plasminogen-activating and inflammation systems. Furthermore, injection of hypoxia-induced angiogenic factors from cultured retinal pigment epithelium (RPE) cells increased iris vascular sprouting in punctured eyes, indicating that puncture-induced iris angiogenesis in the mouse could be used as new neovascularization model with the possibility of direct noninvasive in vivo analysis.

## Material and methods

### Animals

Thirty-six 12.5-day-old (P12.5) BalbC mice of either sex (Charles River, Cologne, Germany) were used in accordance with the statement for the Use of Animals in Ophthalmologic and Vision Research and the study protocols were approved by Stockholm’s Committee for Ethical Animal Research. Mice were housed in social groups with 12 h day/night cycle, free access to food and water, and monitored daily. Euthanasia was performed by cervical dislocation, as approved by the ethical committee.

### Puncture-induced iris angiogenesis

Eighteen mice were anesthetized with 4% isoflurane (Baxter, Kista, Sweden) and iris angiogenesis was induced by puncture of one eye and compared to non-punctured fellow-eye as control. Self-sealing punctures were performed with a beveled 30G needle posterior to the limbal limit of the uvea on both sides of the eye (superior and inferior) and repeated every fourth day. Punctures perforate the uveal tissue between the ciliary body and the ora serrata. Great care was taken to not perforate the lens or alter the intraocular pressure. No observable effects were denoted on the retina nor vitreous body. After each injury, a drop of 1% tetracaine hydrochloride (Bausch & Lomb, Rochester, NY, USA) was applied on the punctured eye and a subcutaneous injection of 0.9% NaCl (Baxter) was administered to rehydrate the animal.

For VEGF-blockage, 6 mice were intravitreally injected with 1 μL of the recombinant VEGF receptor (VEGFR)1 Fc chimera protein (Bio-Techne Ltd., Abingdon, UK) at 1 gL^-1^ as previously described [12], while the fellow-eye was injected with phosphate-buffered saline (PBS; ThermoFisher Scientific Corp) as vehicle. For hypoxia-induced angiogenesis (see RPE medium conditioning), 12 mice were separated into 2 groups. For the first group, one eye was kept as control and the fellow-eye was punctured and injected intravitreally with 1 μL of vehicle. On the second group, mice were punctured in both eyes and injected in one eye with 1 μL of normoxia-conditioned medium while the fellow-eye was injected with 1 μL of hypoxia-conditioned medium. Similarly, punctures and injections were repeated every fourth day. On day 15, mice were euthanized by cervical dislocation and the eyes were enucleated, and extraneous tissues were removed from the eyes.

### Noninvasive in vivo analyses

Mice were anesthetized and, prior to puncture or injections at each experimental day, irises were photographed in vivo using a camera phone (iPhone 4S, Apple Inc., Cupertino, CA, USA) mounted onto an ocular adapter. Irises were selected as a region-of-interest (ROI) in ImageJ (NIH freeware), and in vivo vasculature was determined by grey densitometric mean of an 8-bit image corrected by the total area of ROI.

### RPE medium conditioning

ARPE-19 cells (ATCC, Manassas, VA, USA) were maintained in F12 nutrient mix containing glutaMAX-1 supplemented with 10% fetal bovine serum (FBS), 1% penicillin/streptomycin mix (all media and supplements from ThermoFisher Scientific Corp., Waltham, MA, USA). Confluent cells were changed to reduced medium containing 1% FBS and were maintained 24 h at normoxia in a standard cell culture incubator (20% oxygen, 5% carbon dioxide at 37°C) or exposed to 24 h of hypoxia (1% oxygen, 5% carbon dioxide at 37°C). Media were collected and clarified by centrifugation at 2500 g and stored at -80°C. Reduced medium was used as vehicle. Prior to injection, all media were equilibrated to room temperature (RT) and filtered through a 22 μm pore membrane. Soluble angiogenic factors were analyzed from normoxia- and hypoxia-conditioned ARPE-19 media using a proteome profiler array (Bio-Techne Ltd.), according to the manufacturer’s instructions.

### Immunofluorescence

Twelve eyes (6 controls and 6 punctured) were fixed for 6h at RT in 4% buffered formaldehyde (FA; Solveco, Rosersberg, Sweden) and extensively washed with PBS. Irises were carefully microdissected from the whole eye and prepared for free-floating immunostaining. Irises were permeabilized 15 min with PBS containing 0.5% Triton X-100 (Sigma-Aldrich Corp., St. Louis, MO, USA). Antigen retrieval was performed by microwave-heating for 3 min using Diva decloacker (Biocare Medical, Concorde, CA, USA). Samples were blocked 1 h with blocking buffer (10% normal goat serum from ThermoFisher Scientific Corp., 0.1% Triton X-100 in PBS) at RT. Primary antibody anti-platelet endothelial cell adhesion molecule (PECAM)-1 (cat. no. 562939, BD Biosciences, Bedford, MA, USA) and anti-NG2 chondroitin sulfate proteoglycan (cat. no. AB5320, Millipore, Temecula, CA; USA), and isolectin-biotin (cat. no. I21414, ThermoFisher Scientific Corp.) were incubated overnight (ON) at 4°C, at 1:200 in blocking buffer. Secondary antibodies anti-rat conjugated with Alexa488 and anti-rabbit with Alexa-546, and streptavidin-Alexa350 (cat. no. A11006, A11010 and S11249, ThermoFisher Scientific Corp.) were incubated 1 h at RT, at 1:500 in blocking buffer. Antibody steps were extensively washed with PBS. Post-fixing was performed with FA for 10 min at RT. Irises were mounted in fluorescence medium (Dako, Carpinteria, CA, USA). Images were acquired by fluorescence microscopy using an Axioskop 2 plus with the AxioVision software (Zeiss, Gottingen, Germany).

### Sprouting assay

Using AxioVision images, sprouts were identified as discontinuous PECAM-1–positive blood vessels within the iris vasculature and counted using ImageJ (NIH freeware). Data were analyzed as number of sprouts per total area of each corresponding iris.

### Quantitative PCR

Twenty-four eyes were zap-frozen in liquid nitrogen as two eyes per sample (6 pairs as controls and 6 pairs as puncture) and stored at -80°C until extraction. Total RNA was extracted from both whole-eyes using AllPrep kit (Qiagen, Hilden, Germany), according to the manufacturer’s instructions. Generation of cDNA was performed using 1 μg of RNA by iScript reverse transcriptase (BioRad Laboratories, Hercules, CA, USA). Transcript levels were determined by quantitative real-time RT-PCR (qPCR) using cDNA and iQ SYBR Green Supermix with gene-specific PrimePCR primer-pairs, on a MyiQ qPCR system (all PCR reagents and equipment from BioRad Laboratories). Data were determined by relative transcript expression to two housekeep genes (ΔΔCT method) and normalized to non-punctured controls. Angiogenesis regulated transcripts: VEGFA; VEGFR1 and -2; platelet growth factor (PGF); PAI-1; urokinase-type plasminogen activator receptor (uPAR); matrix metalloproteinases (MMP)2 and -9; interleukins (IL)1β and -6; C-C motif chemokine ligand (CCL)2; and C-X-C motif chemokine receptor (CXCR)4. Housekeep genes: hypoxanthine phosphoribosyltransferase (HPRT); and TATA-box binding protein (TBP).

### Immunoblotting

Twenty-four eyes were used to generate whole-tissue protein extracts using the AllPrep kit, as described (see quantitative PCR). Total protein was separated by SDS-PAGE and transferred onto nitrocellulose membranes. Membranes were blocked with 5% non-fat milk (nfm) in Tris-buffered saline (TBS; BioRad Laboratories). Primary antibodies were incubated ON at 4°C in 1:200 dilutions, while secondary was incubated 1 h at RT as 1:2000 dilution, in 1% nfm-TBS containing 0.05% Tween-20 (TBS-T; Sigma-Aldrich Corp.). Both antibody steps were extensively washed with TBS-T, developed with enhanced chemiluminescence plus reagent (ThermoFisher Scientific Corp.) and exposed to ECL autoradiography hyperfilms (VWR, Lutterworth, UK). Primary antibodies: anti-VEGFA; anti-plasminogen; anti-IL6 (cat. no. ab9570, ab154560, and ab6672, Abcam, Cambridge, UK); anti-CXCR4 (cat. no. NB100-56437, Bio-Techne Ltd.); and anti–pan-Actin (cat. no. A2066, Sigma-Aldrich Corp.). Secondary antibody: horseradish peroxidase-conjugated anti-rabbit immunoglobulins (cat. no. P0399, Dako).

### Statistical analysis

Noninvasive in vivo iris vasculature analyses were determined from 6 mice per group. Total RNA and protein from mice eyes was collected from 12 mice as a pool of 2 independent eyes. Sprouting assay was determined from 6 irises from independent animals. All experiments were repeated at least twice with a final n = 6, and results are presented as mean ± standard error (SEM). Two-tailed Student’s t-test was used for statistical evaluation and P < 0.05 was considered statistically significant. When applicable, multiple comparison test by ANOVA with Bonferroni correction was performed.

## Results

### Uveal puncture induces iris neovascularization

**Fig 1:**
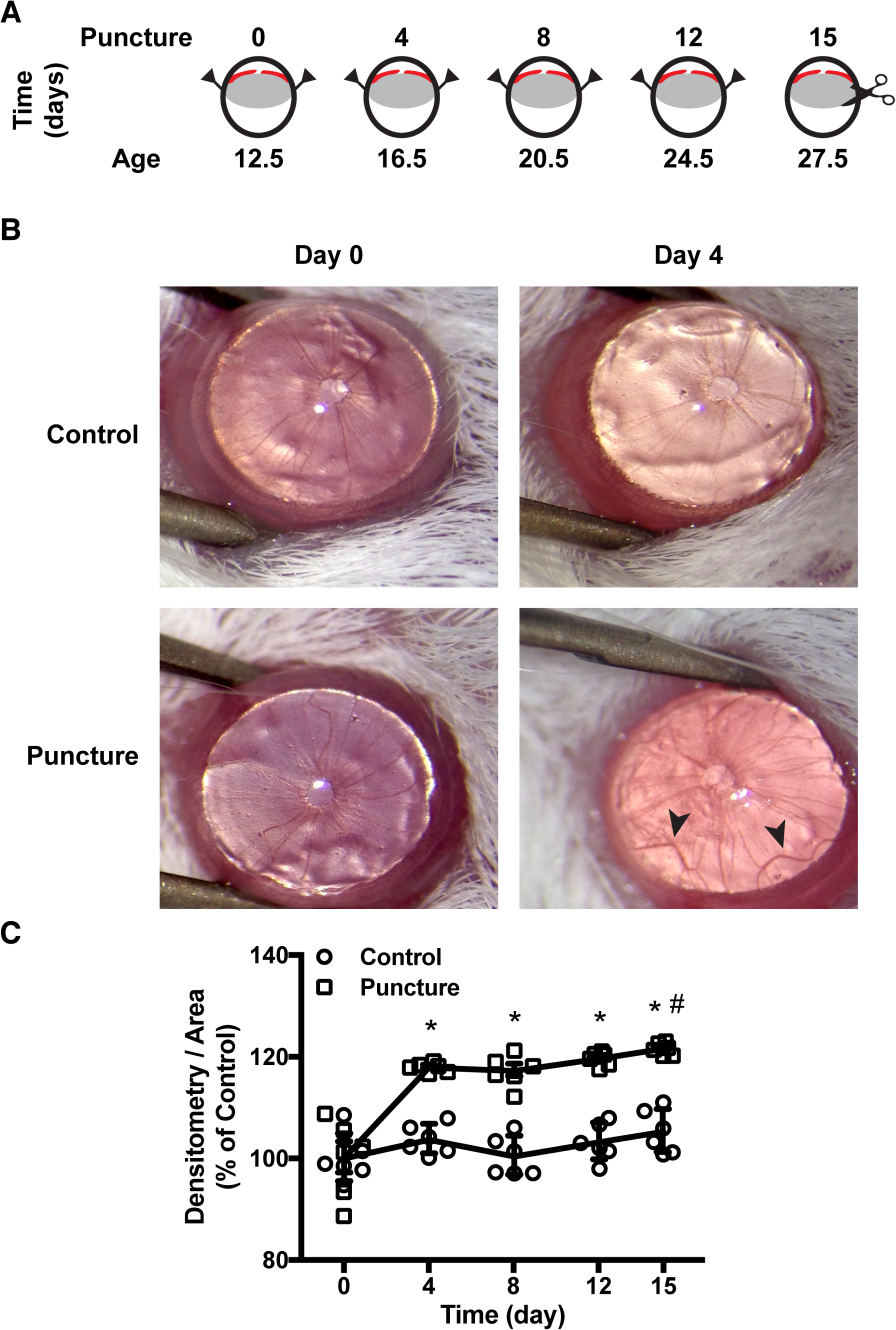
Puncture-induced iris angiogenesis. (**A**) Schematic representation of iris angiogenesis protocol. P12.5 mice were punctured in both sides (arrow). Punctures were repeated every fourth day posteriorly to the iris (red). At P27.5 enucleation was performed and irises were dissected from the eye. (**B**) Pictures of mice eyes at day 0 and day 4 of punctures from control and punctured animals. Vascular loops are shown by arrow on day 4 of a puncture-induced mouse, near the puncture site. (**C**) Quantitative analysis of in vivo iris vasculature by noninvasive methods. Photos of irises were analyzed by densitometric mean corrected by area and determined as percentage of day 0. Data is presented as mean ± SEM of independent irises (n = 6). Multiple comparison test showed a statistical increase of blood vessels in puncture-induced irises (* P < 0.05 versus control, # P < 0.05 versus day 4; two-way ANOVA).

The eye of newborn mice is still undergoing the later stages of ocular development, with pups opening their eyes approximately at P12.5, while ocular development occurs until approximately P40 [3]. Little is known regarding iris vasculogenesis, yet models of OIR suggest vascular development of the retina until P21 [5]. In the present study, BalbC mice pups at P12.5 were subjected to uveal punctures (S1 Video), with repeats every fourth day until P24.5. Fifteen days after the first injection (P27.5), mice were euthanized and irises were dissected for analysis (Fig 1A). Due to the transparency of the cornea and the lack or iris pigmentation of the albino BalbC mice, iris blood vessels could readily be observed during the experimental procedure. An initial observation revealed an increase in vascular loops, predominantly in puncture-induced eyes (Fig 1B), clearly noticeably from day 4 after the initial puncture and sustained during the experimental procedure. These observations suggest that wounding of the uveal tissue by puncture could trigger wound healing, with production of angiogenic factors, inducing iris neoangiogenesis. Moreover, due to the transparency of the cornea, the newly formed iris blood vessels could be observed by noninvasive methods. Using a camera adapted to the surgical stereoscope, images of the mice irises were acquired throughout the experimental days and quantified for density of blood vessels (Fig 1C). A significant increase in blood vessels density was observed at day 4 on puncture-induced irises when compared to control (P < 0.001), and was sustained through the following experimental days. Interestingly, at day 15, a slight yet significant increase (P = 0.002) could be denoted on puncture-induced iris vascular density when compared to day 4.

### Puncture-induced iris neovascularization is characterized by increased sprouting

**Fig 2:**
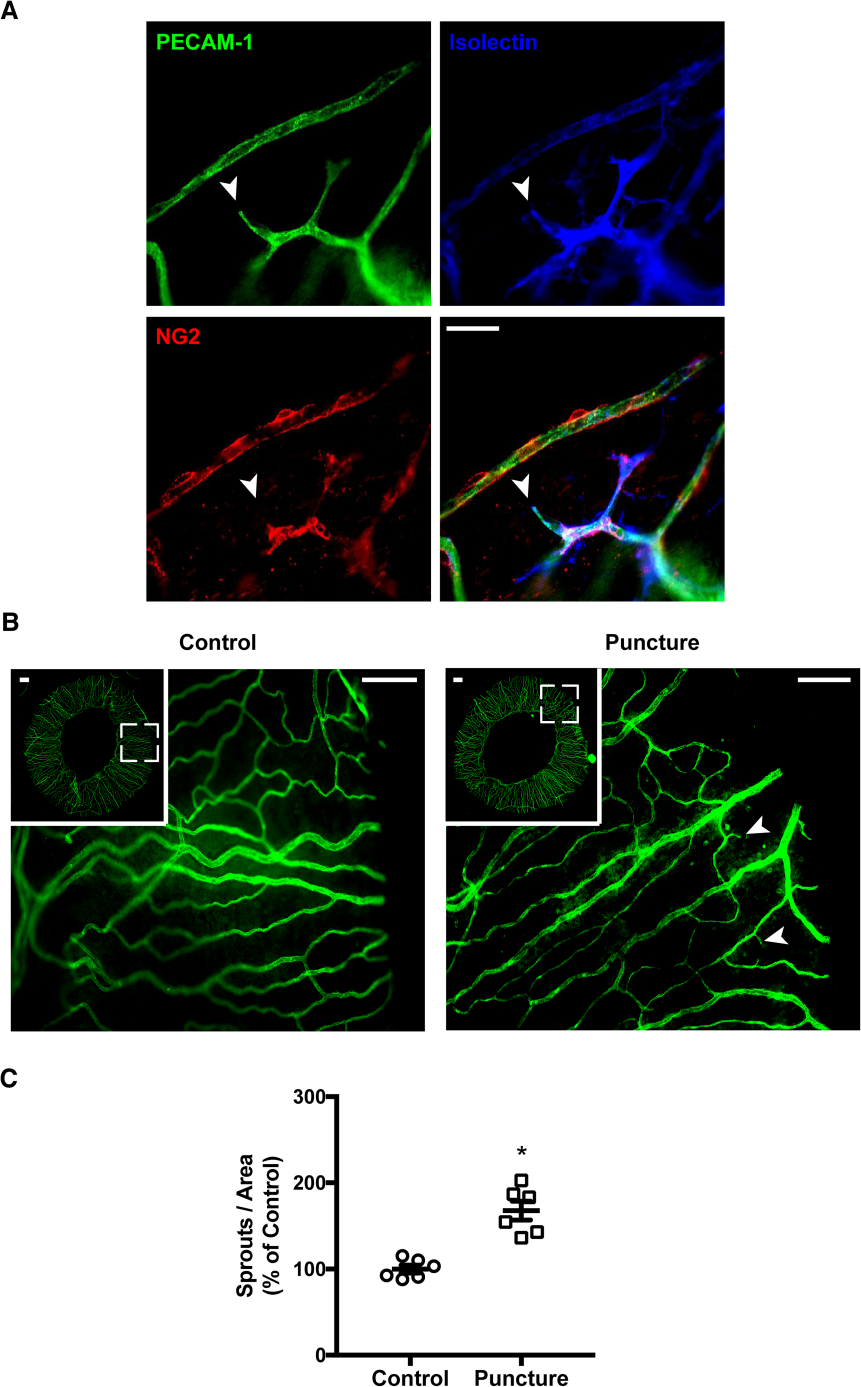
Vascular sprouting in puncture-induced iris neovascularization. (**A**) Irises were immunostained for PECAM-1, isolectin, and NG2. Sprouts were identified as PECAM-1–positive discontinuous vessels, which colocalized with isolectin yet not with NG2 (arrow). Scale bar = 400 μm. (**B**) Irises from control and puncture-induced mice were immunostained for PECAM-1. Sprouts are identified on regional magnifications (arrows). Scale bar = 200 μm. (**C**) Quantification of number sprouts corrected by area and determined as percentage of control. Data is presented as mean ± SEM of independent irises (n = 6). A significant increase in sprouts was observed in puncture-induced eyes (* P < 0.05; two-tailed t-test).

At day 15 mice were euthanized and eyes were enucleated, the eyeballs were incubated in FA for 6h to both fix and increase tissue rigidity. After methodically developing a protocol for microdissection (S2 Video), the mouse irises were carefully transferred to free-floating in PBS. Subsequently, the isolated irises were immunostained with PECAM-1, an endothelial marker, for visualization of whole-mount blood vessels (S3 Video). A subset of irises was co-stained with NG2 and isolectin-IB4, markers for vascular pericytes and endothelial cells respectively, revealing that newly formed vessels were NG2–negative while PECAM-1– and isolectin–positive (Fig 2A). Upon immunofluorescence microscopy analyses, an increase in the number of vascular sprouts was denoted on puncture-induced irises when compare to non-punctured controls (Fig 2B), as depicted by discontinuous PECAM-1–positive blood vessels. After normalization with the total area of the corresponding iris, an increase of 67% of the number of vascular sprouts (Fig 2C) was determined on puncture-induced irises (P = 0.001).

### Plasminogen-activating and inflammation mediate puncture-induced iris neovascularization

**Fig 3:**
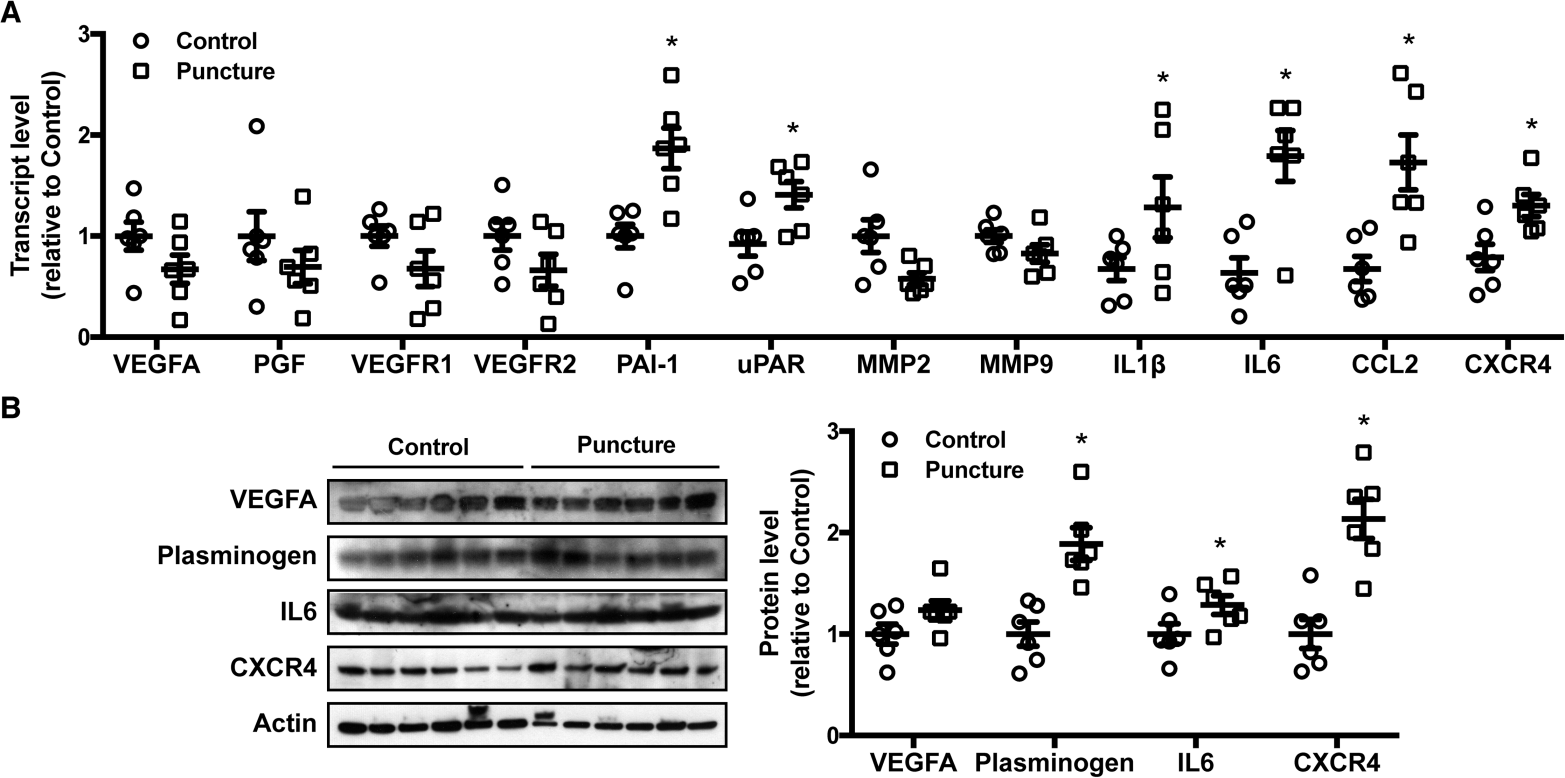
Angiogenesis-related factors in puncture-induced iris neovascularization. (**A**) Transcript levels were quantified by qPCR on puncture-induced mice eyes. Results are presented normalized to control. Data is represented as mean ± SEM of 12 mice as a pool of 2 independent eyes (n = 6). (**B**) Protein levels were determined by densitometric analysis and corrected versus the actin loading control. Data is presented as for qPCR. Inflammation markers and plasminogen-activating genes are significantly increased (* P < 0.05; two-tailed t-test).

To assess the involvement of angiogenic factors during puncture-induction process, qPCR was performed. Postulating that puncture wounding could be upregulating angiogenic factors in tissues such as the uvea which could then trigger iris angiogenesis, whole eyeballs, rather than isolated irises, were used for RNA and protein extraction. Transcript levels were analyzed for a multitude of vascular factors involved in endothelial proliferation, extracellular matrix regulation, and inflammatory molecules related with angiogenesis (Fig 3A). Curiously, genes involved in endothelial proliferation as VEGF factors and its receptors (VEGFR), and genes involved in extracellular matrix degradation (MMPs) did not differ significantly between punctured and control eyes. Genes associated with plasminogen-activating system, namely PAI-1 and uPAR were statistically increased (1.86-fold, P < 0.001, and 1.41-fold, P = 0.037 respectively). Interestingly, all inflammation markers studied (IL1β 1.79-fold, P = 0.01; IL6 1.80-fold, P < 0.001; CCL2 1.73-fold, P < 0.001; and CXCR4 1.36-fold, P = 0.037) displayed a significant increase in puncture-induced eyes when compared to controls. Protein levels were analyzed by immunoblotting of relevant factors and determined by densitometry versus the actin loading control (Fig 3B). Interestingly, VEGFA showed no statistical difference, confirming the qPCR results. A statistical increase (1.89-fold, P = 0.005) in plasminogen is associated with increased PAI-1 transcript levels. Moreover, increased expression of IL6 and CXCR4 (1.27-fold, P = 0.008, and 2.14-fold, P = 0.002) could be confirmed at the protein level. Together, these data suggest a role for inflammatory and plasminogen-activating systems in upregulating the vascular sprouting response observed in the iris of puncture-induced eyes.

### VEGF-blockage partially reduces iris neovascularization

**Fig 4:**
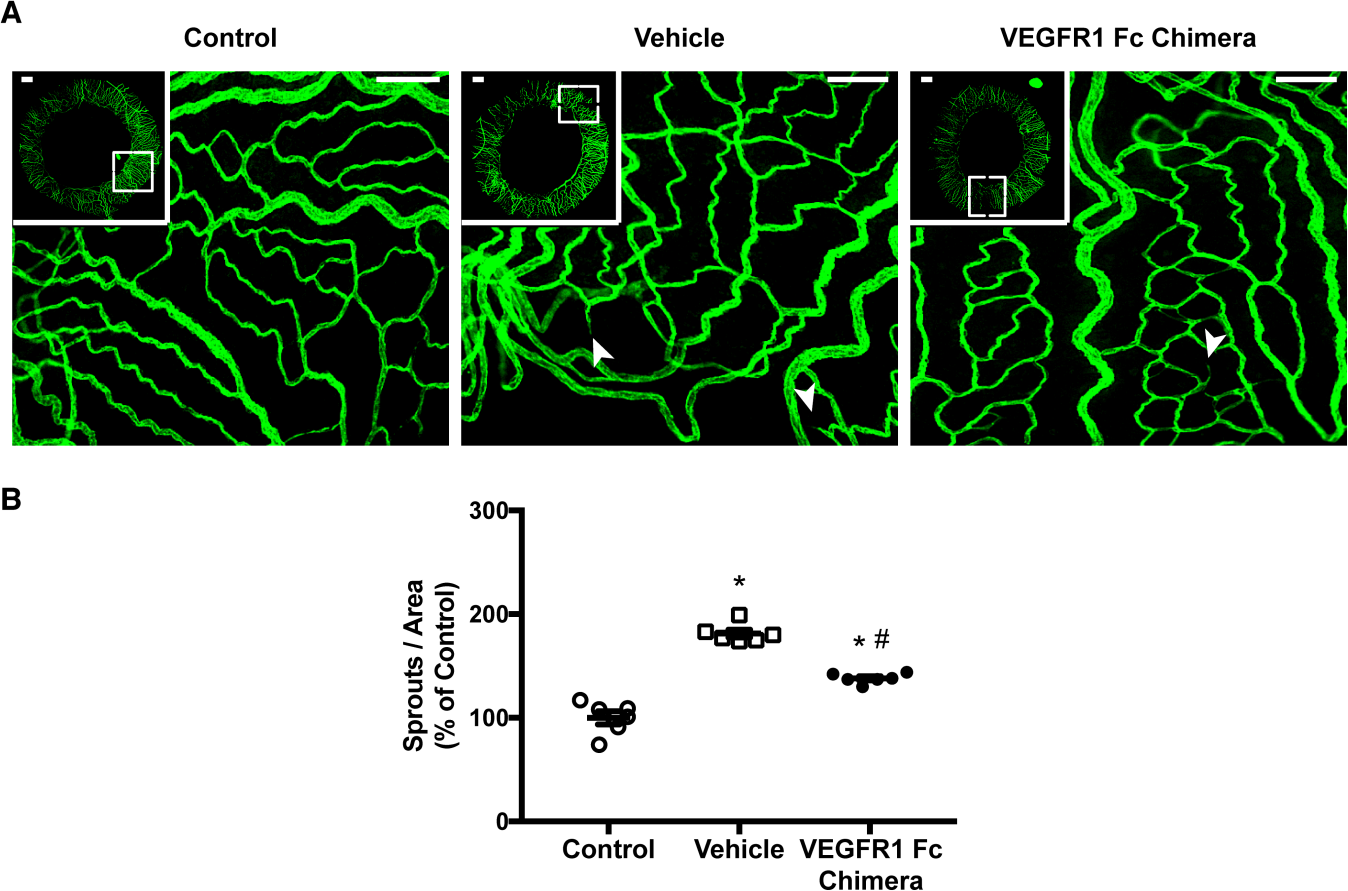
VEGF-blockage in puncture-induced iris neoangiogenesis. (**A**) PECAM-1–positive blood vessels and sprouts were analyzed on irises treated with empty vehicle, or VEGFR1 Fc chimeric protein, a mouse equivalent of aflibercept. Non-punctured eyes were used as control. Arrows denote vascular sprouts. Scale bar = 200 μm. (**B**) Quantitative data are represented as number of sprouts per iris area as percent of non-punctured control (mean ± SEM, n = 6). Multiple comparison test showed a statistical decrease of VEGFR1 Fc chimera treated irises, yet insufficient to reduce to control levels (* P < 0.05 versus control, # P < 0.05 versus vehicle; one-way ANOVA).

Transcript levels of VEGFA and its receptors did not differ significantly, yet a slight increase in protein levels could be observed. Anti-VEGF substances are commonly used in ophthalmology to treat vascular and edema-related pathologies. Consequently, we have treated puncture-induced eyes with a recombinant chimeric protein of VEGFR1 and the Fc fragment of IgG, a mouse equivalent of the clinically used aflibercept (Fig 4). An increase in sprouts on irises of vehicle treated puncture-induced eyes was observed, with a noticeable decrease in number of sprouts on VEGFR1 Fc chimera treated eyes (Fig 4A). Quantitative analysis of relative number of sprouts corrected by the corresponding area (Fig 4B) of puncture-induced irises treated with vehicle showed a near 70% increase (P < 0.001) versus control, a result within the range observed with puncture alone (Fig 2C). Interestingly, VEGFR1 Fc chimera treated eyes were reduced to approximately 40% over control. Albeit treatment with the recombinant chimeric protein significantly reduced relative sprouts when compared to vehicle injected (P < 0.001), it remained significantly higher than control levels (P < 0.001), suggesting that VEGF-blockage could only partially reduce iris neovascularization in puncture-induced eyes.

### Hypoxia-conditioned RPE cells increase iris neovascularization

**Fig 5:**
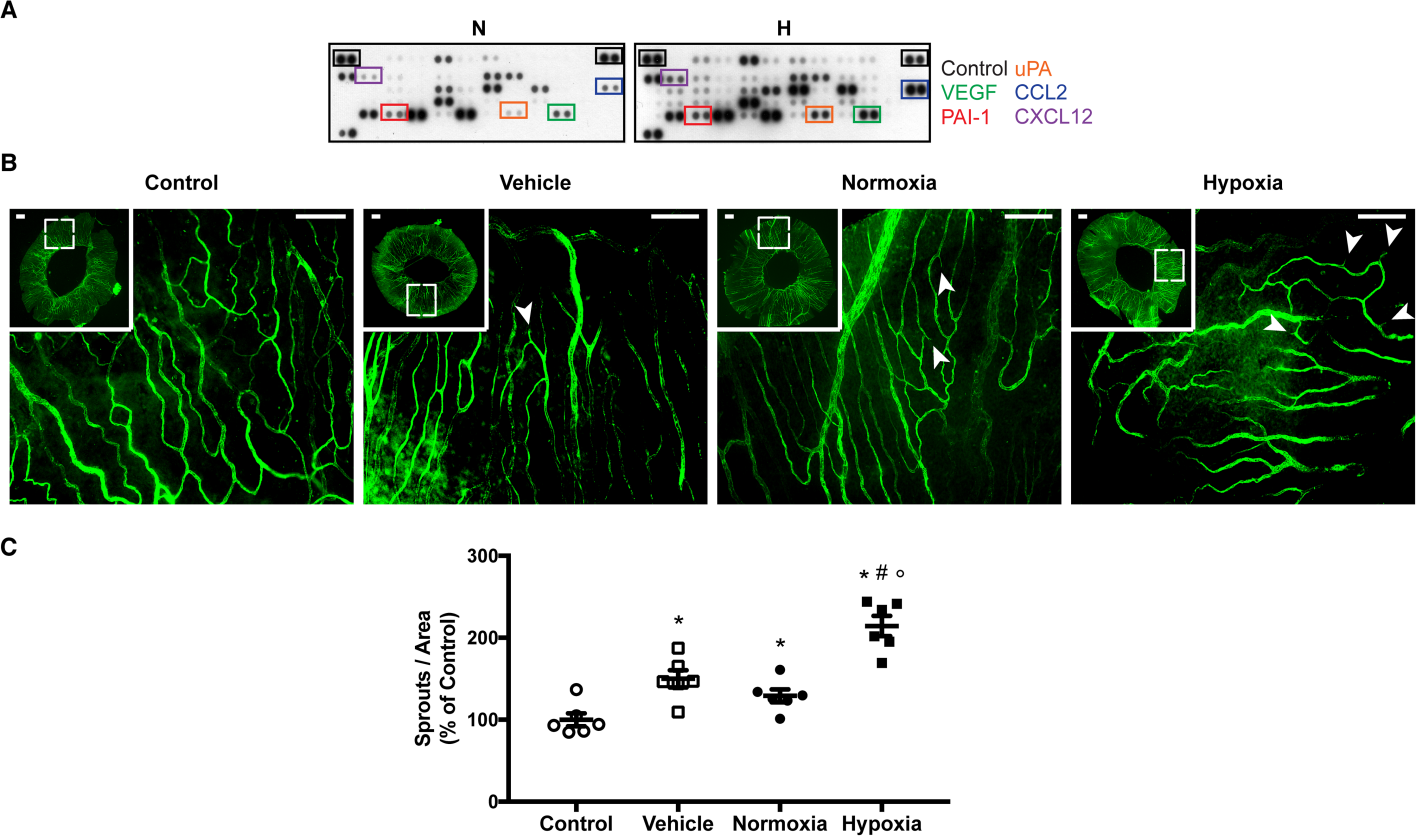
Effects of hypoxia-conditioned media from retinal pigment epithelium cells in puncture-induced iris neovascularization. (**A**) Angiogenic factors of normoxia- or hypoxia-conditioned ARPE-19 media was evaluated by proteome array. Blots with equivalent exposures are presented for best comparison, as depicted by the levels of positive controls’ expression. A general increase in angiogenic factors is denoted upon hypoxia-conditioning, with relevance on iris puncture-induced associated factors. (**B**) PECAM-1–positive blood vessels and sprouts were analyzed on irises treated with empty vehicle, or normoxia- or hypoxia-conditioned medium from RPE cells. Non-punctured eyes were used as control. Arrows denote vascular sprouts. Scale bar = 200 μm. (**C**) Quantitative data are represented as number of sprouts per iris area as percent of non-punctured control (mean ± SEM, n = 6). Multiple comparison test showed a statistical increase of hypoxia-conditioned treated irises (* P < 0.05 versus control, # P < 0.05 versus vehicle, ° P < 0.05 versus normoxia; one-way ANOVA).

Hypoxic exposure of RPE cells has been shown to increase hypoxia-inducible factors (HIF) protein level and transcriptional activity [13]. Consequently, hypoxia can upregulate expression of angiogenic factors such as VEGF, uPAR, IL6, and CCL2 [14–17]. As such, we exposed ARPE-19 cells to normoxia or hypoxia for 24 h and collected the conditioned media as a source of multiple angiogenic factors. A generalized increase in angiogenic factors could be observed by proteome arrays in hypoxia-conditioned media from ARPE-19 cells when compared to normoxia-conditioning (Fig 5A), particularly in levels of VEGF, PAI-1, uPA (uPAR ligand), CCL2, and CXCL12 (CXCR4 ligand), factors directly associated with puncture-induced iris neovascularization (Fig 3). Subsequently, puncture-induced eyes were injected with RPE conditioned media, and iris neovascular response was determined by relative sprout numbers as previously. Expectedly, an increase in number of sprouts in punctured eyes was observed, with a noticeable increase in eyes treated with hypoxia-conditioned RPE medium (Fig 5B). Quantitative analysis was performed as for VEGF-blockage and the relative number of sprouts (Fig 5C) of puncture-induced irises treated with vehicle showed 50% increase (P = 0.002) versus control, again within the range observed with puncture alone (Fig 2C). Curiously, normoxia-conditioned medium did not statistically differ from vehicle treated irises, yet displaying a significant increase of approximately 30% (P = 0.013) when compared to control. Interestingly, eyes treated with hypoxia-conditioned ARPE-19 medium showed a significant increase of 114% in relative number of sprouts in iris vasculature as compared to non-punctured controls (P < 0.001), and above 50% when compared to vehicle or normoxia-conditioned medium (P = 0.001 and P < 0.001, respectively). These findings indicate that hypoxia-exposed RPE cells medium can exacerbate puncture-induced iris neovascularization by increasing intravitreal concentration of multiple angiogenic factors.

## Discussion

Angiogenesis in the adult is generally associated with multiple physiologic events where dynamic regulation of vasculature is necessary, including wound healing. Nevertheless, angiogenesis is also associated with several pathologies comprehending tumors and infarctions, amongst others. In the eye, a number of sight-threatening conditions are directly associated with an imbalanced production of angiogenic factors, culminating in severe vision loss [18]. In some ocular pathologies, as PDR and neovascular glaucoma [9], the imbalance in soluble angiogenic factors is sufficient to develop clinical iris pathoangiogenesis, ie. rubeosis iridis. Fundamentally, these ocular diseases demonstrate a plasticity of the adult iris to undergo neoangiogenesis, illustrating that a mouse model of iris induced angiogenesis could be achieved by promoting an imbalance in angiogenic factors in either or both anterior and posterior chambers of the eye. In fact, previous studies have shown that injection of bioactive VEGF into the vitreous of nonhuman primates could lead to an exacerbated iris angiogenic response [10].

In the present study, evidence of increased iris angiogenesis after a puncture to the uveal tissue is demonstrated for the first time. Ocular vascular development in mice is estimated to continue postpartum [1,3,4]. Postulating that young mice pups would respond preferably to angiogenesis, a series of uveal punctures were performed during the ocular developmental period, after the eye lids were naturally opened. These punctures would trigger wound healing mechanisms and increase angiogenic factors accordingly with tissue repair. The most important characteristic of the anterior segment of the eye is transparency. This transparency results of a particular collagen structure and an absence of vascularization. Accordingly, a direct visualization of the iris is possible and angiography can be performed to visualize the blood vessels [9,10]. In the case of albino BalbC mice, the lack of pigmentation in the iris allows for direct visualization of the blood vessels. Upon uveal wounding, formation of vascular loops was observed, with increased incidence in puncture-induced eyes. This observation suggests that in vivo noninvasive assessment and quantification of iris induced vasculature can be achieved easily with direct visualization or with high-resolution imaging methods, as has been previously proposed for the anterior chamber of the eye [19]. When analyzed by immunofluorescence, at day 15 post-puncture induction, small vessel sprouts where observed. Markers for vascular pericytes and endothelial cells revealed that the newly formed vessels were NG2–negative while PECAM-1– and isolectin–positive. Animal models of induced sprouting angiogenesis in adult have determined pericyte recruitment as early as 7 days after induction of the model [20,21]. The finding of NG2–negative vascular sprouts in the presented iris model could be in agreement with ocular vascular development at P27.5, where new blood vessels, possibly anastomosis between arteries and veins, are still being formed. Nonetheless, we cannot exclude that evaluation of puncture-induced iris neovascularization model could be preferable at earlier time points, depending on specific experimental conditions.

Molecular analysis of angiogenesis-related genes in puncture-induced eyes revealed an increase in the plasminogen-activating system through PAI-1 and uPAR transcripts and plasminogen protein levels, together with cytokines such as IL1β, IL6 and CCL2, and the chemokine receptor CXCR4. Interestingly, the particular combination of PAI-1 activating uPAR and cytokines activating chemokine receptors has been reported to upregulate vasculogenesis in endothelial cells in a study of high-throughput screening on vascular development in animal models [22], while VEGF and growth factor-mediated signaling would be preferentially associated with angiogenesis. In fact, adult nonhuman primates exposed to increased intravitreal VEGF displayed clear signs of iris angiogenesis [10], yet in the presented model the vascular events observed in the iris seem better associated with increased ocular vascular development in puncture-induced irises. Little is known about iris vasculogenesis and angiogenesis, yet anastomosis between arteries and veins is characteristic of this tissue [1]. The findings that the plasminogen-activating and inflammatory systems were upregulated while the VEGF system remained unaltered in this model of uveal puncture-induced iris neovascularization could suggest that the vascular sprouting increase observed in puncture-induced irises could be linked to development of vascular anastomosis rather than canonical neoangiogenesis. Such might be an effect of the young age of the animals used in this study, when ocular vascular development is still present, and could account for the presence of sprouts observed in non-punctured eyes. In contrast with the adult animals used in the nonhuman primate studies, where VEGF was injected intravitreally to induce iris angiogenesis [10], the puncture-induced iris angiogenesis in young mice presented an unaltered VEGF system, which suggests that classical inhibition of VEGF, as is customary in ophthalmology, might be ineffective in this method or produce only partial effects from crosstalk between VEGFRs and uPAR [16].

Consequently, VEGF-blockage was performed with a mouse specific anti-VEGF chimeric protein, an equivalent of the clinically used aflibercept. Interestingly, VEGF-blockage could only partially reduce iris neovascularization. These findings are in agreement with the molecular analysis of the puncture-induced eyes, where VEGFA was non-significantly regulated. Nevertheless, anti-VEGF treatment significantly reduced puncture-induced iris vascularization, suggesting that tissue-specific levels of VEGFA in the iris could still be regulated by anti-VEGF, and illustrates the potential of the presented iris neovascularization model with regard to anti-angiogenic treatments.

Exposure of RPE cells to hypoxic environment has been shown to lead to increased transcription and expression of several angiogenic factors, including growth factors, plasminogen-activating system molecules, cytokines, and chemokine receptors [13–16]. These factors have been associated with iris neovascularization, both in the present study and others [10,17,22]. Consequently, treatment of puncture-induced irises with hypoxia-conditioned medium from ARPE-19 cells showed a markedly increase in iris neovascularization, and could even be suggested as a neovascular glaucoma mouse model. These data, together with VEGF-blockage, confirm that uveal puncture is a bonafide model of iris neovascularization and that this model could be used to assess the role of molecules with pro- and/or anti-angiogenic effects.

In sum, this study demonstrates a novel angiogenic method by uveal puncture with subsequent iris neovascularization in mice. Furthermore, the current model has been characterized as a wound healing response with increased expression of the plasminogen-activating and inflammatory systems as angiogenic factors. Moreover, the iris induced neovascularization mouse model has been shown to respond by exacerbated neovascularization when treated with angiogenic factors, indicating its use in molecular studies of angiogenesis-related molecules. Finally, the transparency of the anterior segment of the eye allows for a direct noninvasive visualization of iris neovascularization, facilitating evaluation and quantification of the newly formed blood vessels in vivo.

## Supporting information

S1 Video Uveal puncture. (MOV)

S2 Video Iris dissection. (MOV)

S3 Video Iris flat-mounting. (MOV)
